# The Regulatory Function of CCR9^+^ Dendritic Cells in Inflammation and Autoimmunity

**DOI:** 10.3389/fimmu.2020.536326

**Published:** 2020-10-02

**Authors:** Manisha Pathak, Girdhari Lal

**Affiliations:** Laboratory of Autoimmunity and Tolerance, National Centre for Cell Science, Pune, India

**Keywords:** C-C chemokine receptor type 9 (CCR9), Foxp3^+^ Regulatory T cells, CCL25, dendritic cell (DC), mucosal tolerance

## Abstract

Chemokine receptor CCR9 is a G protein–coupled receptor and expressed on several types of immune cells, including dendritic cells (DCs), CD4^+^ T cells, and B cells. CCR9 drives the migration of immune cells to gradients of its cognate ligand CCL25. The chemokine CCL25 is mostly produced by gut and thymic epithelial cells. Gut- and thymic-homing DCs are known to express CCR9, and these cells are predominantly localized in the gut lining and thymus. CCR9^+^ DCs are implicated in regulating inflammation, food allergy, alloimmunity, and autoimmunity. Differential interaction of CCR9^+^ DCs with lymphoid and myeloid cells in the thymus, secondary lymphoid tissues, and mucosal sites offer crucial insights to immune regulation. In this review, we examine the phenotypes, distributions, and interactions of CCR9^+^ DCs with other immune cells, elucidating their functions and role in inflammation and autoimmunity.

## Introduction

Chemokine receptor CCR9 has important homeostatic and regulatory functions and drives the migration of immune cells to the gradient of CCL25 (also known as thymus-expressed chemokine, TECK). Expression of CCR9 is reported on the majority of gut-homing CD4^+^ and CD8^+^ T cells, gamma-delta T cells, plasmacytoid dendritic cells (pDCs), IgA plasmablast, IgA plasma cells, and intraepithelial lymphocytes (IELs) (1–3). Thymic and intestinal epithelial cells constitutively express CCL25 in mice and humans (4), and it is overexpressed in the intestine during gut inflammation and autoimmunity (1, 5–9). Due to the upregulation of CCL25 and recruitment of CCR9^+^ immune cells in the gut of inflammatory bowel disease (IBD) patients, CCR9 is considered as a potential therapeutic target to control gut inflammation (1, 10, 11). However, Ccr9^-/-^ or Ccl25^-/-^ mice show increased severity of dextran sodium sulfate (DSS)-induced colitis (12). Ccr9^-/-^ mice have very low capacity to induce immune tolerance to oral antigens (5). In parallel, clinical trials with CCR9 antagonist CCX282-B have been disappointing and display dose-dependent adverse reactions in Crohn’s disease (10, 11, 13). Further, it is shown that CCR9 expression in CD4^+^ T cells in gut inflammation has a dispensable function (14).

On the other hand, DCs play an important role in maintaining gut homeostasis and inflammation in mice and humans. DCs in mouse lymphoid organs can be broadly classified into two groups: conventional DCs (cDC; B220^-^CD11c^hi^) and pDCs (B220^+^CD11c^int^). Using flow cytometry and CyTOF, multidimensional analysis of DCs in the different tissues in humans and mice is been elegantly characterized and shows a heterogeneous population of DCs than just cDCs and pDCs (15). A study in 2009 by a group of researchers led by Villandangos show that both DC subsets differ in their developmental and functional properties (16). The DCs are known to exhibit inflammatory and tolerogenic functions in the gut. Gut DCs are present within the gut-associated lymphoid tissues (GALTs), which include Peyer’s patch (PP) and solitary isolated lymphoid tissues (ILT) or even distributed throughout lamina propria (LP) (17). Gut-homing DCs are further classified into four subsets based on the expression of the surface markers CD103 and CD11b; CD103^+^CD11b^-^, CD103^-^CD11b^+^, CD103^+^CD11b^+^ and CD103^-^CD11b^-^ (18). CD103^+^CD11b^−^ falls under cDC1, whereas CD103^+^CD11b^+^ forms subgroup cDC2 (15, 19, 20). CCR9 and other gut-homing chemokine receptors drive the migration of DCs into the GALTs. The localization of DCs in secondary lymphoid tissues, and its interaction with immune cells meticulously tunes the balance between homeostasis and inflammation. In this review, we discuss how CCR9^+^ DCs regulate the phenotype and function of innate and adaptive immune cells during homeostasis and tolerance.

## Distribution and Function of CCR9^+^ DCs

**Figure 1:**
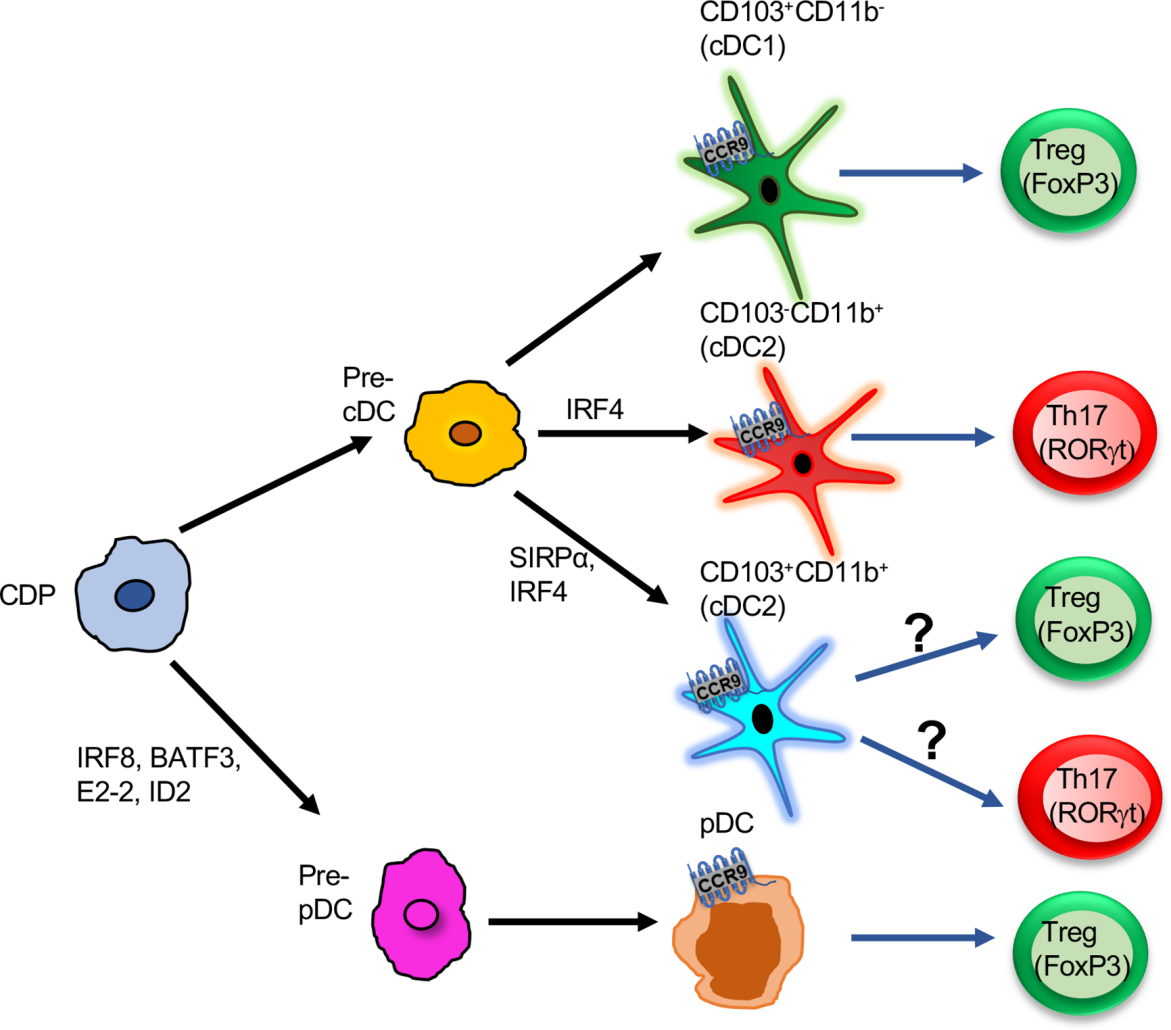
Functions of CCR9^+^ DCs in the intestine: Pre-pDCs or pre-cDCs derived from CDP progenitors. Further pre-cDCs are differentiated into three subsets based on the expression of surface markers CD103 and CD11b: CD103^+^CD11b^-^, CD103^-^CD11b^+^, CD103^+^CD11b^+^. All three subsets express CCR9 and induce either T cells to differentiate into Tregs or Th17. CD103^+^CD11b^-^ is the potent inducer of Treg cells. Pre-pDCs differentiate into pDCs, and CCR9^+^ pDCs promote Treg differentiation in the gut. CDP, Common dendritic cell progenitor; Pre-cDC, pre-common dendritic cell; Pre-pDC, pre-plasmacytoid dendritic cell; cDC, common dendritic cell; Treg, T regulatory cells; pDC, plasmacytoid dendritic cell.

In the gut, DCs are present in mesenteric lymph node (mLN), LP, and PP to mount an effective immune response (21, 22). The mucosal tolerance induction by DCs to innocuous antigens by promoting Tregs and producing sIgA in the intestine are discussed earlier (22). However, during inflammation, DCs promote the generation of Th1/Th17 by secreting proinflammatory cytokines (23). CCR9 controls the migration of CD11c^+^ DCs into the gut (14, 24) and also drives the recruitment of various subsets of DCs into the PP and mLN during inflammation (14). PP with specialized M cells uptake and recognize particulate antigens in a controlled manner and induce tolerance during homeostasis (25, 26). In colitis, many DCs are recruited into the subepithelial dome. These DCs internalize the bacteria and translocate into the PP (27). CD103^+^ DCs play a protective role at the initial phase of inflammation, whereas in the chronic phase, CD11b^+^ DCs show a pathogenic role by inducing Th1/Th17 response (28, 29). Our recent study demonstrates that CCR9 is expressed in various subsets of DCs during homeostatic and gut inflammation (14). Inflammation increases CCR9 expression on CD103^+^CD11b^-^, CD103^-^CD11b^+^, and CD103^+^CD11b^+^ subset in PP and mLN (14). Thus, CCR9 affects the distribution of a different subset of cDCs in the gut during homeostasis and inflammation (**Figure 1**). A study from Wendland et al. reveals that CCR9 controls the migration of pDCs into the gut under homeostasis and inflammation. Furthermore, these intestinal pDCs help in the rapid mobilization of myeloid DCs into LP (8). In addition to distribution, CCR9 affects the function of DCs during inflammation (14, 24). Previous work has shown that CCR9^+^ pDCs inhibit T cell proliferation and induce Foxp3^+^ regulatory T cells (7). However, the intrinsic mechanism of CCR9 signaling that controls the expression of costimulatory and regulatory molecules on cDCs and its effects on the distribution and function of DCs in the GALTs require detailed investigation.

## Role of CCR9^+^ DCs in the Induction of Central Tolerance

The role of DCs in inducing peripheral and central tolerance is well known (7, 30, 31). In thymus, DCs have extrathymic and intrathymic origins and are very heterogeneous. In the thymic medulla, DCs and medullary thymic epithelial cells (mTEC) express MHC-I and MHC-II and act as very important antigen-presenting cells. The negative selection of αβ TCR cells and Foxp3^+^ Treg development requires direct recognition of self-antigens *via* MHC class II present on mTEC and thymic DCs (32–34). DCs can take up antigens from the peripheral tissues and migrate into the thymus, thus playing a role in controlling the development of Foxp3^+^ natural Tregs (nTregs) (35). CCR2 and CCR9 are crucial chemokine receptors involved in the homing of DCs in the thymus (31, 36). CCR9 is expressed at an early developmental T cell stage (double negative 3; DN3 stage), during which thymocytes undergo β selection (rearranging of the TCR beta chain expression along with the pre-T alpha chain) (37). The successful β selection leads the thymocytes to enter the DN4 stage and become CD4^+^CD8^+^ thymocytes and then further undergo positive and negative selection. In the thymic microenvironment, thymic stromal cells express chemokine CCL25 and CCL2 and control the migration of thymic DC and control the central tolerance (4, 36). Thymic cDC2 expressing CCR2 and Ccr2^-/-^ mice show defective negative selection (38). pDC in the thymus expressing CCR9 and Ccr9^-/-^ mice show a defect in the migration of pDC in the thymus as well as impairment in thymocyte deletion (31). It has been reported that CCR7 drives the recruitment of cDCs in the thymus as Ccr7^-/-^, Ccl21a^-/-^, or Ccl19^-/-^ mice that show a defect in the migration of cDC progenitors (39). CCL2/CCR2 interaction helps in the migration of cDCs into the thymic cortex and localizing them to perivascular spaces where they further participate in central tolerance by depleting autoreactive T cell clones (36, 38, 40). This homing process is also controlled by lymphotoxin α (LTα), which negatively regulates CCL2, CCL8, and CCL12 chemokines in the thymus (40). CCL8 is also a ligand for CCR1 and CCR5 and involved in the migration of pDCs and cDCs in the thymus (40). Our recent study also suggests that CD103^+^ DCs and thymic DCs are a potent inducer of Treg in the presence of CCL25 (14). Thus, chemokine receptors play an important role in the thymic settling of DCs and controlling the central tolerance.

## Molecular Mechanism of CCR9^+^ DCs in Inflammation and Autoimmunity

Upon antigen encounter, various signaling pathways, such as JAK/STAT3, Wnt/β-catenin, and AKT/mTOR pathways, get activated in DCs, altering gene expression (41). STAT3 and MAP kinase signaling activate IL-10, TGF-*β*, and aldehyde dehydrogenase (ALDH), which, in turn, induces tolerance, and any disruption in these pathways leads to loss of T cell tolerance and cause gut inflammation (41–43). However, little is known about the molecular mechanism of how CCR9 affects DC function and their phenotype. Only a few studies have attempted to address this issue and provide preliminary insights into the underlying molecular mechanisms in DCs. DCs are well-known antigen-presenting cells, and CCR9 signaling DCs has an inverse relation in DC maturation (24). V-ATPase is known to play an important role in homeostasis and disease. V-ATPases are affected by activation through toll-like receptor signaling, glucose, and amino acid availability in the microenvironment (44). PI-3 kinase and mTOR signaling is known to upregulate DC maturation by assembling the domains of V-ATPase. Consequentially, inhibition of the V-ATPase domain assembly may affect the antigen processing and presentation in the DCs and promoting tolerogenic phenotype (45). Previous studies report that the NF*κ*B pathway prevents the transcription of proinflammatory genes and promotes the tolerogenic DC phenotype. NF*κ*B is designated as a critical marker of TSLP production in airway epithelial cells (46, 47). As a prelude to unraveling the functional and phenotypic insights under the homeostasis condition, we show that CCR9^hi^ DCs have a lower costimulatory molecule and show thymic-stromal lymphopoietin (TSLP)-mediated regulation of the immune response (14). Based on these recent investigations, we suggest that it could be possible that CCR9 may use this mechanism to prevent the maturation of DCs by inhibiting the V-ATPase domain assembly or activating the NF*κ*B pathway to regulate TSLP secretion. In either case, it may contribute to the tolerogenic function of DCs (45, 46). Thus, we comment that, apart from these two possible mechanisms, CCR9 may regulate other signaling pathways in DCs, contributing to tolerogenic function in the gut, which is an interesting area of research requiring further investigation. In the next sections, we critically review the potential role of CCR9^+^ DCs in regulating the CD4^+^ T cell, B cell, and innate lymphoid cell responses.

### Regulation of Different Subsets of CD4 T Cells

As discussed above, inflammation induces the expression of CCL25 to several folds in intestinal epithelial cells and drives the recruitment of various subsets of DCs (14, 48). Apart from the chemotactic role of CCR9 to CCL25, we follow up in our recent study to show how its intrinsic signaling in DCs affects the differentiation of regulatory Foxp3^+^ CD4^+^ T cells (Tregs) (14). We recall that CCR9^+^ DCs are present in the mLN and PP, promoting Tregs’ differentiation, whereas CCR9^-^ DCs drive the Th17 cells in the presence of CCL25 (14). These studies indicate that CCR9^-^ DCs are more inflammatory in the phenotype and drive the differentiation of naïve CD4^+^ T cells into Th1/Th17. Intriguingly, CCR9^+^ DCs show reduced expression of costimulatory molecules (MHC II and CD86) and increased expression of regulatory molecules such as FasL and latency-associated peptides (LAP) (14, 49, 50). A study from the Blanchard group in 2009 shows that higher expression of CCR9 inhibits IL-2 production, causing apoptosis of T cells and promoting tolerance in mice (24). In contrast, the low or absent CCR9 on bone marrow (BM)–generated DCs in the presence of GM-CSF increases the expression of inflammatory molecules, which, in turn, induces proliferation and expansion of T cells (24). However, it is reported that BM treated with GM-CSF gives rise to macrophages (51). A study from Wurbel et al. shows that the CCR9^+^ macrophage responds to CCL25 gradient and displays proinflammatory and anti-inflammatory functions (48). Therefore, further studies are required to understand how CCL25/CCR9 interaction regulates macrophage function. The different subsets of DCs show diverse functions in the intestine by expressing various surface molecules and cytokines (41). CD103^+^ DCs mediate tolerogenic function (52) while CD11b^+^ DCs regulate inflammatory responses by producing IL-12, IL-23, iNOS, and TNF-α (53). Our recent study shows that CD103^+^CD11b^-^ DCs promote Treg differentiation in the presence of CCL25 (14). TSLP is highly expressed by CD103^+^ DCs, which promote the differentiation of Foxp3^+^ Tregs by directly interacting with its receptor on CD4^+^ T cells or limiting their potential to drive Th1 cells (54). These studies suggest that CCR9^+^CD103^+^ DCs are the most probable promoters of Treg induction *via* secreting TSLP molecule while CCR9^+^CD11b^+^ DCs induce the Th1/Th17 response by expressing proinflammatory cytokines (14, 20, 55). We comment that it could be possible that, during inflammation, CD11b^+^ DCs lose CCR9 expression due to altered gene expression and promoting proinflammatory response. Nonetheless, the role of TSLP in the presence or absence of CCL25 in DCs require further investigation.

### Regulation of B Cell Response

**Figure 2:**
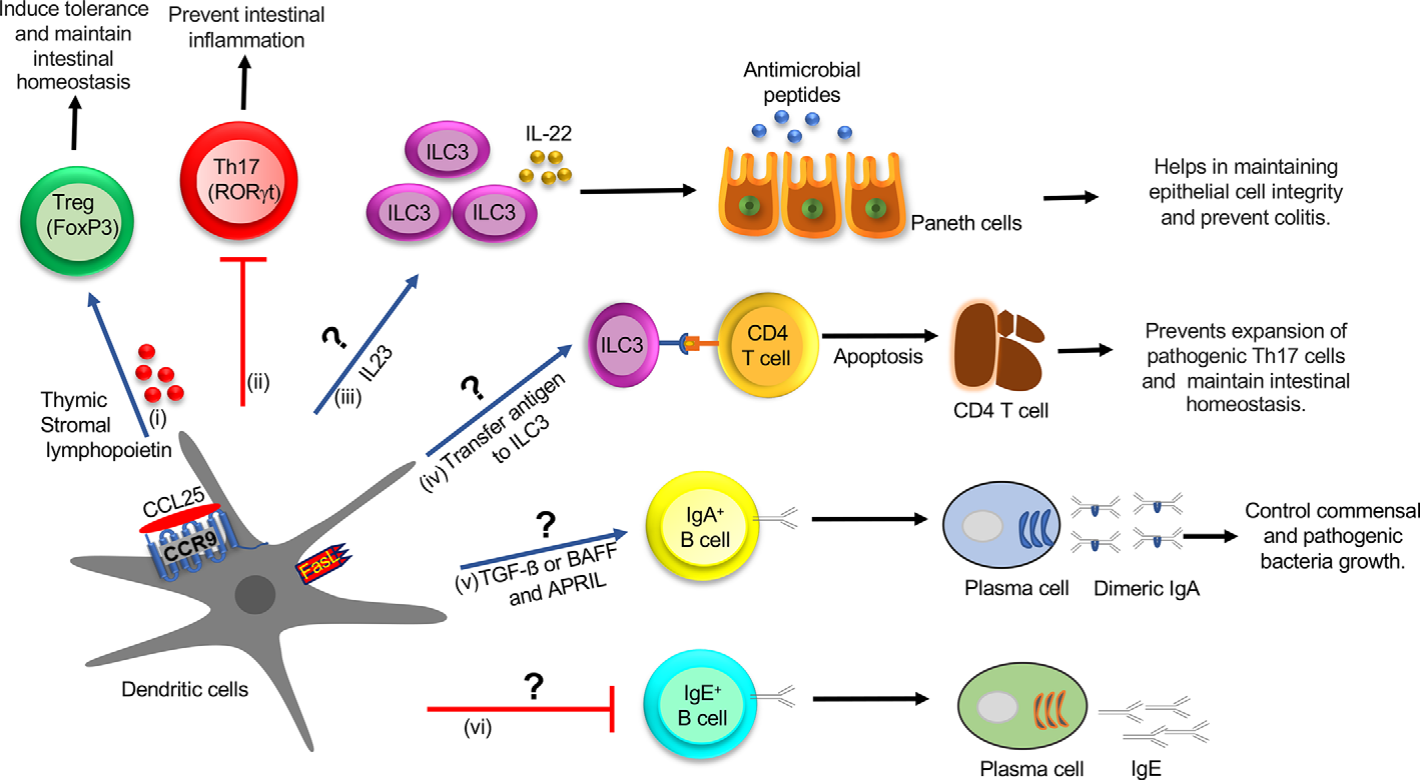
Role of CCR9^+^ DCs in the regulation of innate and adaptive immune cell function in the intestine during homeostasis: (i) CCL25-CCR9 interaction on DCs increase the production of TSLP and expression of FasL and LAP, which promotes Treg differentiation, which induces tolerance and maintains intestinal homeostasis. (ii) CCR9^+^ DCs inhibit Th17 differentiation by an unknown mechanism, which prevents intestinal inflammation. (iii) CCR9^+^ DCs might regulate the ILC3 function by regulating IL-22 production by secreting IL-23 cytokine. IL-22 augments the production of AMP and helps in maintaining epithelial cell integrity and preventing colitis. (iv) ILC3 may acquire antigens from CCR9^+^ DCs and eliminate commensal-reactive CD4^+^ T cells by activating the apoptotic pathway, which prevents the expansion of pathogenic Th17 cells and maintains intestinal homeostasis. (v) CCR9^+^ DCs may regulate IgA^+^ B cell class switching by activating the TGF-β pathway or increasing expression of BAFF or APRIL and generate high-affinity IgA antibodies, which control commensal and pathogenic bacteria growth. TSLP provides an autocrine effect on DCs and increases expression of BAFF or APRIL, which help in IgA class switching. (vi) CCR9^+^ DCs may inhibit IgE class switching by an unknown mechanism. DC, Dendritic cell; Treg, T regulatory cells; TSLP, thymic stromal lymphopoietin; LAP, latency-associated peptide; ILCs, innate lymphoid cells; AMP, antimicrobial peptide; BAFF, B-cell activating factor; APRIL, A proliferation-inducing ligand.

The incoming antigens into the GALTs are sampled by DCs that reside just beneath the subepithelial dome (SED) region underlying the follicle-associated epithelium (FAE) (25). This local sampling of antigens by DCs in the PP established by studies so far is believed to be critical to the induction of adaptive mucosal immunity (56, 57). On the other hand, IgA class switching occurs in both a T cell–dependent and –independent manner (58). Tolerogenic DCs, therefore, trigger the inductive and effector phase of the IgA response in a T cell–dependent route in the PP (57, 58). DCs are known to offer antigens to CD4^+^ T cells in the perifollicular region of PP or B cell in the SED, which, in turn, activates the TGF-β pathway and promotes IgA class switching and generates high-affinity IgA antibodies (57). These DCs further help in the migration of the plasma cell precursor to LP by upregulating the expression of gut-homing receptors, α4β7-integrin and CCR9 (59). In the T cell–independent pathway, epithelial cells trigger DCs to increase the expression of both B-cell activating factor (BAFF) and a proliferation-inducing ligand (APRIL), which promotes IgA class switching (60). TSLP also provides an autocrine effect on DC and increases expression of BAFF or APRIL, which is required for IgA class switching in the intestine (**Figure 2**). In addition, BAFF and APRIL are also critical regulators of the IgE-specific class-switch recombination (CSR) in the presence of IL-4 (61). On the other hand, our study elucidates that the adoptive transfer of CCR9^+^ DCs in an ova-allergy model reduces the IgE response (14) and marginally increases IgA^+^ B cells in the PP and mLN. The presence of cytokines other than TGF-β is known to induce IgG or IgE class switching over the IgA class. With our recent studies in hand, we hypothesize one alternative to the above previously proposed mechanism, i.e., CCR9^+^ DC inhibits IL-4 production, which activates B cells toward IgA switching over to IgE. However, further mechanistic details of how CCR9^+^ DCs regulate B cell class switching needs allied investigation and is currently beyond the scope of this review.

### Regulation of Innate Immune Cells

From the preceding sections, it is clear that the role of CCR9^+^ DCs in regulating innate immune cell distribution and function in the intestine currently suffers from poor characterization. Two independent research teams led by Pizzaro and Artisin 2015 in concert show that DCs and innate lymphoid cells (ILCs) cross-talk with each other to maintain gut tolerance, and any perturbation in this cross-talk leads to gut inflammation and colitis (62, 63). On the other hand, ILCs are divided into three subsets. ILC1 is regulated by the transcription factor T-bet and produces cytokines IFN-γ and TNF-α (64). The second group, ILC2, is controlled by transcription factors GATA-3 and Bcl11b and produces Th2 cytokines (65, 66). The third group, ILC3, depends on transcription factor RORγt, and secretes IL-17 and IL-22 cytokines (67). ILC1 and ILC3 play pathogenic roles and are implicated in the epithelial and LP compartment of a mouse model of IBD (62, 63). It is shown that ILC1 is increased in patients with Crohn’s disease (CD), whereas ILC2 is increased in patients with ulcerative colitis (UC) (68). However, IBD patients with established UC and CD have increased frequency of ILC1 and ILC2 (68). It is shown that functional cross-talk between human DCs and ILCs occurs across the lymphoid and nonlymphoid (mucosal) tissues (69). DCs regulate the function of ILCs by producing various cytokines, such as IL-23 and IL-1β (62, 70). TLR5 activation on DCs augments IL-23 production, which induces ILC3 to produce cytokine IL-22 (70). Consequentially, IL-22 helps in maintaining epithelial cell integrity by inducing the production of antimicrobial peptides (AMPs), such as regenerating islet-derived protein 3 beta (RegIII‐β) and RegIII‐γ and mucins from epithelial cells (71). The absence of IL-22 increases Th17 cell expansion and promotes colitis in mice (71). In contrast, ILC3 also regulates IL-22 production by activating lymphotoxin (LT) signaling, which contributes to the development of lymphoid follicles (LF) in the gut (72). In the LF, the interaction of ILC3 and DCs through lymphotoxin beta receptor (LTβR) signaling controls the IL-22 synthesis in ILCs (72). In addition, IL-1β regulates the release of Csf2 by ILC3, which promotes the secretion of retinoic acid (RA) and IL-10 from DCs and macrophages to generate homeostasis in the gut (73). ILC3 acquires antigen from CD103^+^ DCs in LP and eliminates commensal-reactive CD4^+^ T cells in the mLN during homeostasis (74). ILC3 T cell interaction inhibits IL-2 production and induces apoptosis of effector CD4^+^ T cells (74). However, in IBD, this function is compromised due to the low expression of MHC-II on ILC3, which governs the expansion of pathogenic Th17 cells (74). Hepworth et al. shows that ILC3 and thymic epithelial cells show regulation of MHC-II expression, and MHC-II^+^ ILC3s can directly induce cell death in activated commensal bacteria-specific CD4^+^ T cells (75). Together, these studies indicate that CCR9^+^CD103^+^ DCs may induce suppressor function in T cells directly by secreting TSLP and indirectly *via* regulating MHC-II expression on ILC3 and during inflammation. We suggest that ILC3 may acquire antigens from other subsets of CCR9^+^ DC, which perturbs the MHC-II presentation of ILC3 and induces a Th17 response. In conclusion, ILCs are crucial determinants of pathogen immunity and intestinal homeostasis. Nonetheless, the mechanism of CCR9^+^ DC regulation by ILCs in the intestine during colitis remains a stone unturned and requires further investigation.

## Future Perspectives

Our recent study in the mouse model shows that CCR9^+^ DCs contribute to controlling the intestinal inflammation by regulating innate and adaptive immune responses (14). CCL25-CCR9 is studied mostly as a homing receptor. Like another gut-tropic chemokine receptor CCR6 intrinsic signaling, known to alter the phenotype and function of CD4 T cells (76), how the intrinsic signaling of CCR9 manipulates the phenotype and function of DCs is not known. In this review, we focus on the inevitable role of CCR9 in the migration of DCs and how it affects its function during gut inflammation. This review spells out that further studies are indispensable to define intrinsic molecular and cellular signaling of CCR9 in various subsets of DCs. Such studies are expected to offer new pathways to control intestinal inﬂammation and autoimmunity. Future studies with specific deletion of CCR9 in the subsets of DCs during intestinal inﬂammation will throw more light on its importance under both homeostatic and inflammatory conditions.

## Author Contributions

MP and GL wrote the manuscript. All authors contributed to the article and approved the submitted version.

## Funding

MP received the Research Associate fellowship from Science and Engineering Research Board, Ministry of Science and Technology, Government of India (PDF/2015/000792). GL received grants from the Department of Biotechnology, (Grants numbers, BT/PR15533/MED/30/1616/2015; BT/PR14156/BRB/10/1515/2016), Swarnjayanti Fellowship (DST/SJF/LSA-01/2017-18) from Department of Science and Technology, Ministry of Science and Technology, Government of India.

## Conflict of Interest

The authors declare that the research was conducted in the absence of any commercial or financial relationships that could be construed as a potential conflict of interest.
